# Examen Tu Salud: A Digital Spiritual Health Intervention for Young Adult US Latinas

**DOI:** 10.1007/s10943-025-02270-1

**Published:** 2025-02-21

**Authors:** Kelly L’Engle, Adam Landeros Cavazos, Evelin Trejo

**Affiliations:** School of Nursing and Health Professions, University of San Francisco, 2130 Fulton Street, San Francisco, CA 94114 USA

**Keywords:** Spirituality, Latina, Young adults, Digital intervention, Coaching, Mental health, Health and wellness

## Abstract

Although spiritual health is a core dimension of health and wellness, particularly for Latinos, it receives limited attention in health promotion interventions. *Examen Tu Salud* is a brief intervention for young Latinas in the USA and is designed using culturally tailored spiritual messaging and education provided through daily multimedia messages and weekly remote peer coaching rooted in Ignatian values and pedagogy. Results from this single group intervention study showed that after four weeks, participants reported large increases in spiritual health (Cohen *d* = 0.82), well-being (Cohen *d* = 0.91), and happiness (Cohen *d* = 0.84), and moderate reductions in stress (Cohen *d* = 0.41) and anxiety (Cohen *d* = 0.49). These findings further develop the spiritual health intervention literature and establish a baseline for future brief digital health interventions to support Latinas and other groups using culturally tailored spiritual health messaging.

## Introduction

Across the lifecourse, religion and spirituality are powerful determinants of health (Chen et al., 2020). Both spirituality and religiosity are associated with better physical health outcomes for communities around the world, including better outcomes associated with diabetes and cardiovascular disease, and overall lower mortality (Chen et al., 2020; George et al., 2000; Koenig, 2012). People identifying as spiritual or religious report lower rates of depression and anxiety (Chen et al., 2020; George et al., 2000) and an increased ability to manage stress and other mental health concerns (Koenig, 2012). Systematic reviews show that religious and spiritual interventions help to reduce stress, depression, and alcoholism (Goncalves et al., 2015; Marques et al., 2022) and may improve quality of life, physical activity, weight control, and other health behaviors (Derose & Rodriguez, 2020; Goncalves et al., 2017; Lancaster et al., 2014). Health practitioners are increasingly incorporating spirituality into traditional intervention approaches (Goncalves et al., 2015; Marques et al., 2022). For example, adaptations of diabetes prevention programs that incorporate faith-based texts and spiritual messages into health curricula have led to weight loss and healthier eating among Latino and African American participants (Derose et al., 2019; Gutierrez et al., 2014).

Latinos use spirituality and religion to cope with serious illnesses such as arthritis, depression, and breast cancer, and spirituality itself is a source of resilience and thriving for Latinos during stressful times (Hunter-Hernandez et al., 2015; Morgan Consoli et al., 2018). Approximately half of the US Latino population identifies as Catholic, 92% report a belief in God, 75% partake in daily or weekly prayer practices, and 73% say faith is very important in their life (Barna Group, 2019; Pew Research Center, 2014). Among Latino families, meditation and personal reflections are familiar (Pew Research Center, 2014) and typically practiced in the home and local community, particularly among Catholics (Barna Group, 2019). Many Latinos rely on religious and spiritual values in their daily lives and spirituality is an important cultural value (Campesino & Schwartz, 2006; Hunter-Hernandez et al., 2015; Jurkowski et al., 2010).

In fact, Latinos view health as consisting of essential physical, mental, and spiritual aspects which need to be maintained and balanced for overall wellness (Jurkowski et al., 2010). Taking a holistic perspective on health is not only culturally relevant but also may improve participants’ engagement with health programs (Jurkowski et al., 2010; Schwingel & Galvez, 2016). Research with US Latinos and African Americans suggests that faith-based health promotion messages stimulate more active engagement with content than traditional messages (Best et al., 2016; Holt et al., 2008) and that participants in faith-based health programs are motivated to enroll and participate (Krukowski et al., 2010) because the holistic approach and direct links between physical and spiritual health are relevant and tailored to their cultural values (Schwingel & Galvez, 2016; Tettey et al., 2016).

Despite calls over many decades for greater application of spiritual health components into health promotion programs, there are limited examples of these initiatives (Gerhardt-Strachan, 2022; Hawks et al., 1995), especially for Latinos (Derose & Rodriguez, 2020). The 2020 US Census showed that Latinos are the largest and youngest minority group, and Latino youth have some of the highest rates of obesity and prediabetes among all racial/ethnic groups (Katz et al., 2021). Programs that are designed in accordance with Latinos’ distinct cultural values are critically needed for young Latinos. Health promotion interventions have been provided in partnership with religious and faith-based organizations in the US for over a century, particularly in Black/African American communities (Campbell et al., 2007; Levin, 2016), such as offering sermons and Bible-based teaching that encourage healthy lifestyles and nutrition education and cooking classes for congregants (Derose et al., 2019; Gutierrez et al., 2014; Tettey et al., 2016). However, programs for young people today are limited and the research base for Latinos is less developed than for other communities of color (Derose & Rodriguez, 2020; Lancaster et al., 2014). As health professionals seek culturally tailored, effective approaches to prevent and manage chronic diseases, digital technology-supported interventions may be especially promising for engaging and improving health and wellness among minorities in the USA (Gershkowitz et al., 2021; Gonzalez et al., 2021).

*Examen Tu Salud* is a brief digital health intervention designed to improve the physical, mental, and spiritual health of 18 to 29-year-old Latinas (L’Engle et al., 2023). Spiritual health was integrated into wellness messages and coaching strategies to engage and resonate with the cultural values, norms, and concerns of young Latinas and to provide motivation for better self-care via religious and spiritual values. The intervention was evaluated in a single group intervention study design. This paper reports on observed improvements from baseline to follow-up on quantitative measures of spiritual health and mental health with results from in-depth interviews conducted on program completion to further explore how the spiritual components of the intervention contributed to better health and wellness for participants.

## Methods

### Recruitment and Informed Consent

Between January and March 2021, Latina volunteers were recruited from an urban university in Northern California to participate in the 4-week pilot study. Promotional fliers were shared via electronic communication with Latina students and Latina-focused student organizations. A lottery for three $75 gift cards to the bookstore was offered as an incentive for participation. Prospective participants who expressed interest in the study were screened for inclusion and considered eligible to participate if they identified as Latina and female, were between 18 and 29 years old, and owned a mobile phone. A total of 34 participants (83% of prospective participants) were enrolled in the study. The Institutional Review Board for the Protection of Human Subjects at the university approved all study procedures.

### Data Collection

Participants completed an electronic survey at baseline. Approximately one week after the final coaching session and five weeks after completing the baseline survey, participants completed the follow-up electronic survey and an in-depth interview via videoconference to obtain program feedback. The interview guide asked participants their opinions on what aspects of the program were the most useful or provided new information, their favorite digital messages and whether they read or shared messages multiple times, and how their thoughts on health and wellness have changed since starting the program.

### Intervention Design

*Examen Tu Salud* (Examine Your Health) is a four-week program that was co-designed with young adult Latinas. Social Cognitive Theory (SCT, Bandura, 1986) and the Transtheoretical Model (TTM, Prochaska & Velicer, 1997) provided the behavioral intervention framework. The program provided four 30-min weekly coaching sessions via videoconference. Coaches were Latina peers educated in public and behavioral health who were trained to use a coaching guide to implement evidence-based coaching practices that were adapted from an existing health coaching curriculum (Bodenheimer & Ghorob, 2014) and based on motivational interviewing principles (Simmons & Wolever, 2013). Participants simultaneously received a standardized, automated daily text or multimedia message each morning for four weeks. Messages were sent on a fixed schedule, were the same for all participants, and were designed to be stand-alone messages promoting physical, mental, and spiritual health that did not require participant response. Digital messages were developed based on existing curricula (CDC, 2012) and health behavior change theory (SCT, TTM).

Ignatian tradition, values, and pedagogy informed the approach to coaching and digital health and wellness messages. The Ignatian Examen is a practice of intentional and routine reflection for spiritual growth in the form of meditation, prayer, and contemplative reflection (Traub, 2008); intervention messages and coaching prompts encouraged these routine practices. Ignatian values require care for the whole person in mind, body, and spirit, and thus offer a holistic perspective on health and well-being where spirituality is prioritized. Ignatian pedagogies promote action and goal setting for personal growth, reordering priorities, and evaluation of both the present and the future to achieve spiritual health (Chubbuck, 2007). Spiritual growth proceeds by seeking help and connecting with others, being mindful, remembering and integrating successes, and practicing gratitude (Silf, 2007; Traub, 2008). These action-oriented principles of Ignatian tradition, values, and pedagogy complement behavior change theory (SCT, TTM), provide motivation for healthier behavior, and demand critical and collaborative learning. The *Examen Tu Salud* program encouraged reflection and spiritual health through directed coaching questions asked to each person (e.g., What are your goals around spiritual health?) and digital messages that encouraged reflection, gratitude, and promoted mindfulness, prayer, meditation, and other wellness activities for all participants. Coaching questions and digital messages promoting spiritual health were designed specifically for the *Examen Tu Salud* intervention to reflect the concerns, common practices, and language of young Latinas. Table 1 shows examples of digital messages sent to participants that focused on spiritual health and well-being.

**Table 1:**
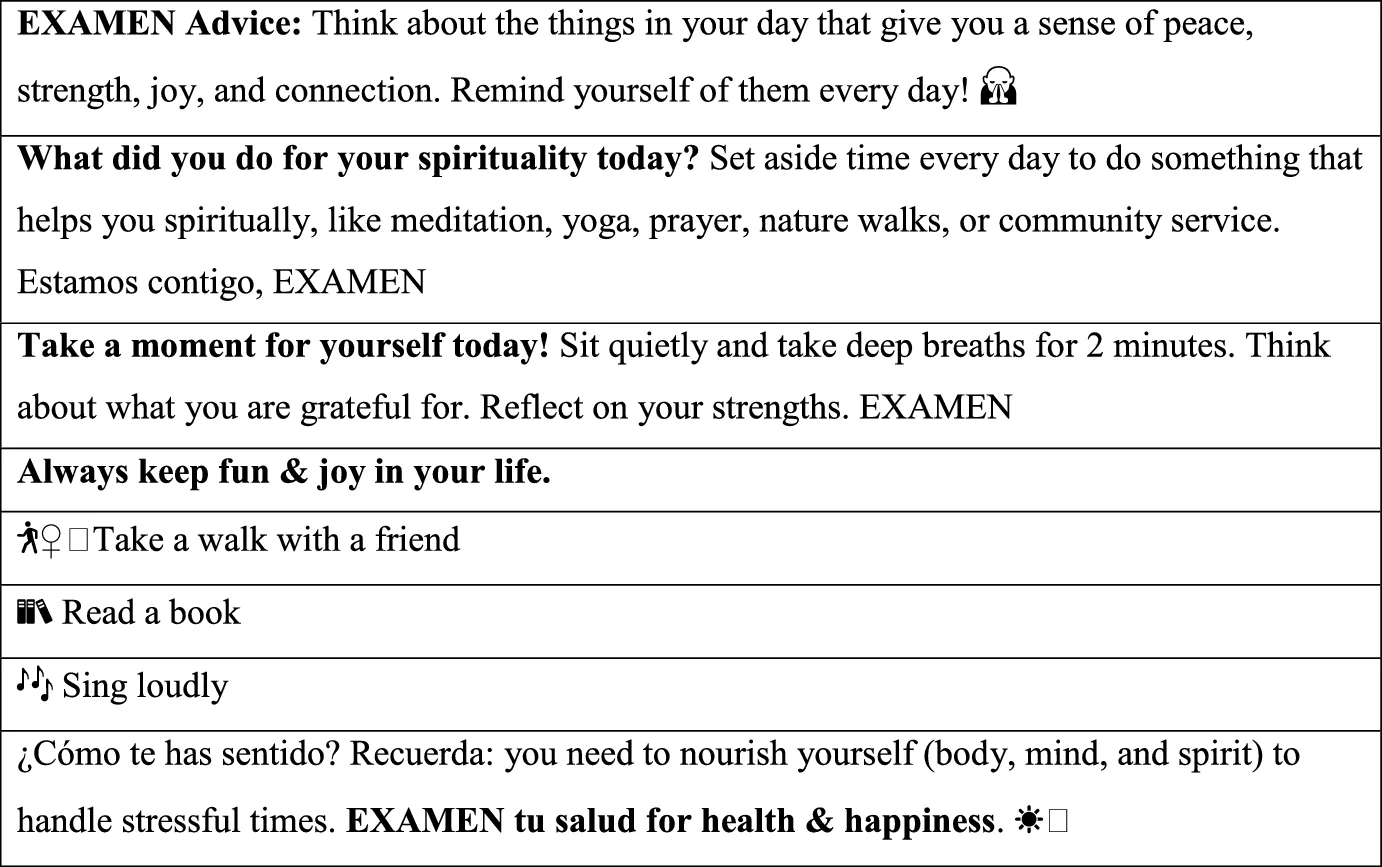
Examples of digital messages on spiritual health and well-being

Ignatian pedagogical principles of acknowledging the individual’s context and lived experience (Chubbuck, 2007) drove intervention design elements toward cultural tailoring. To support intervention development, four focus groups with young adult Latinas from the study community were conducted to learn about mental and physical health concerns and obtain ideas for health promotion and culturally appropriate digital messaging approaches. Thematic analysis (Braun & Clarke, 2006) of the focus group data by the project team identified that 1) healthy eating, physical activity, weight management, stress reduction, mental health, self-care, spirituality, and relationships were priority concerns, and 2) communications that are friendly and positive, feature culturally relevant foods and activities, include Spanish words and phrases, and that include a digital component were viewed as motivational and engaging (L’Engle & Trejo, 2019). Participants’ Latina identity, student-life context, and potential health concerns were addressed through individualized peer coaching and culturally tailored messages. Coaches were of similar age and ethnic background and also recently enrolled in higher education. As shown in Table 1, digital messages incorporated Spanish words, cultural foods, familial values, and culturally relevant images. Cultural tailoring engages participants (Gonzalez et al., 2021) and aligns with Ignatian principles in recognition of participants’ unique cultural identity.

### Measures

#### Spirituality and Religiosity

Spirituality is generally conceptualized as encompassing four domains: connections to the transcendent, self, others, and nature (Gomez & Fischer, 2003; Michaelson et al., 2021). Participants were assessed on these elements of spiritual health using items and scales previously implemented and shown to be valid and reliable for adolescents and young adult samples (Campesino & Schwartz, 2006; Campesino et al., 2009; Chen & VanderWeele, 2018; Gomez & Fisher, 2003; Michaelson et al., 2021; Rew & Wong, 2006). Participants indicated their level of agreement with each question on a 5-point Likert scale from strongly disagree to strongly agree. Items were summed and averaged to create the scale score in each spirituality domain at baseline and follow-up, with higher scores representing greater spiritual health. Appendix 1 includes a complete version of the spiritual health questions.

Connection to the divine or transcendent was assessed with eight items (baseline ⍺ = 0.85, follow-up ⍺ = 0.81) from the Latino Spiritual Perspectives subscale assessing personal relationship with God or a higher power (Campesino & Schwartz, 2006). Example items are: “My spirituality helps get me through bad times,” “I feel close to God or a higher power,” and “God/higher power is loving and kind.” Spiritual well-being, or connection to self, was assessed with three items (baseline ⍺ = 0.74, follow-up ⍺ = 0.79) assessing sense of life purpose and satisfaction (Malinakova et al., 2017): “I believe there is some real purpose for my life,” “I feel good about my future,” and “I feel very fulfilled and satisfied with my life.” Happiness in the last seven days also was assessed and rated on a 4-point scale from not happy at all to very happy.

Connections to others were assessed with three items (baseline ⍺ = 0.76, follow-up ⍺ = 0.83): “I maintain meaningful and fulfilling relationships with others,” “I have a good support network or caring people,” and “I praise other people for their achievements.” Connections to the natural environment were assessed with three items (baseline ⍺ = 0.79, follow-up ⍺ = 0.71): “I engage in environmentally friendly behaviors, like conserving water, recycling, composting, using reusable water bottles, and taking public transportation when feasible,” “I take long walks or hikes to explore and appreciate the environment,” and “I am aware of the earth’s natural resources and their respective limits, and that we need to conserve these resources.”

Religiosity is typically assessed as the practice and importance of religion in one’s life (Chen & VanderWeele, 2018; Koenig, 2012; Morgan Consoli et al., 2018). Participants were asked how often they prayed or meditated alone in the past seven days on a 4-point scale from nearly every day to not at all. Participants also rated the level of importance of religion in their lives on a 4-point scale from very important to not at all important. Values were reverse coded so higher scores indicate increased religiosity.

#### Mental Health

Participants answered four PHQ-4 questions (Patient Health Questionnaire for Anxiety and Depression, Kroenke et al., 2009) reporting if they have been bothered by feeling nervous, anxious, or on edge; feeling down depressed or hopeless; had little interest or pleasure in doing things; or were not able to stop or control worrying over the past seven days. Items were assessed on a 4-point scale from nearly every day to not all, and they were summed and averaged to create the scale score with robust reliability at baseline (⍺ = 0.80) and follow-up (⍺ = 0.87). Higher values indicate less stress and better mental health. Participants also were asked how often they felt stress over the past seven days, assessed on a 4-point scale from nearly every day to not at all. Problems sleeping in the last seven days were indicated on a 5-point scale from all the time to never. Higher values on these measures represent better mental health.

### Data Analysis

Quantitative and qualitative data analyses were conducted to evaluate results from the pilot study for the 31 participants who completed follow-up data collection. Paired sample *T*-tests, confidence intervals around mean changes, and intervention effect sizes (Cohen’s *d*) were calculated using SPSS (Version 28) to assess changes from baseline to one-month follow-up. Interpretation of effect sizes followed convention (Cohen, 1988) with 0.2 considered a small effect, 0.5 moderate, and 0.8 or greater a large effect. Statistical significance was defined as *p* < 0.05, although findings reaching *p* < 0.10 also are reported given the nascent stages of this research. In-depth interviews were imported into NVivo (Version 12) qualitative analysis software, and text queries (e.g., spirituality, prayer, religion) were used to identify comments related to spiritual health. Thematic analysis explored how culturally tailored coaching and messages promoted spiritual health and influenced physical and mental health outcomes. The analysis followed the process outlined by Braun and Clarke (2006): reviewing data, generating initial codes and searching for themes, assigning data to themes, iteratively revising themes and recoding data as new insights arose, and summarizing and interpreting findings. Steps in thematic analysis were continuously discussed within the research team, and if disagreements arose, they were settled by consensus.

## Results

### Sample Characteristics

Participant characteristics at baseline are presented in Table 2. On average, Latina participants were 24.5 years old (standard deviation = 3.1). Most were unmarried (88%) and lived with their parents (47%) or a partner or spouse (24%). The large majority (79%) reported speaking Spanish moderately or very well. Two-thirds (68%) reported their current religion was Catholic, although slightly more than half (56%) said they rarely or never attended religious services. At study enrollment, 10 students (29%) were thinking about or planning to make changes in their health behaviors, while 22 students (65%) were actively working toward better health.

**Table 2:** Baseline characteristics of Latina participants (*N* = 34)

Characteristics	N (%)
*Age categories*	
18–24	16 (47.1)
25–29	18 (52.9)
Never been married	30 (88.2)
*Living situation*	
With parents or other family members	16 (47.1)
With partner or spouse	8 (23.5)
With roommates	5 (14.7)
Alone	4 (11.8)
Finds it difficult to live on present income	8 (23.5)
Speaks Spanish well	27 (79.4)
Catholic	23 (67.7)
*Religious service attendance*	
Once or twice a month	2 (5.9)
A few times a year	13 (38.2)
Rarely	13 (38.2)
Never	6 (17.6)
*Health stage*	
Not interested in pursuing a healthy lifestyle	–
Thinking about making changes	7 (20.6)
Planning on making changes within 30 days	3 (8.8)
Some changes but trouble following through	22 (64.7)
Has had a healthy lifestyle for years	2 (5.9)

### Quantitative Analyses

Spiritual health increased significantly from baseline to follow-up on all four domains, as seen in Table 3. Paired sample *t*-tests showed large improvements in participants feeling a stronger relationship with God or a higher power (*p* < 0.001, *d* = 0.82) and in their spiritual well-being (*p* < 0.001, *d* = 0.91). Moderate increases in feeling connected to others were observed (*p* = 0.003, *d* = 0.54), although feelings about connections to nature increased only a small amount (*p* = 0.078, *d* = 0.26).

**Table 3:** Changes in spiritual health from baseline to follow-up

	*N*	Baseline Mean (*SD*)	Follow-up Mean (*SD*)	Mean change (95% CI)	*d*
Connection to divine	31	3.42 (0.62)	3.72 (0.61)	0.30 (0.17, 0.44)***	0.82
Spiritual well-being	31	3.79 (0.64)	4.33 (0.54)	0.55 (0.32, 0.77)***	0.91
Connections to others	31	4.19 (0.62)	4.59 (0.50)	0.39 (0.12, 0.65)**	0.54
Connection to nature	31	4.10 (0.68)	4.22 (0.59)	0.12 (− 0.05, 0.28)^tt^	0.26
Importance of religion	31	2.32 (0.83)	2.52 (0.93)	0.19 (0.02, 0.37)*	0.41
Prayer in last 7 days	31	2.06 (1.06)	2.35 (1.20)	0.29 (0.07, 0.65)^tt^	0.30
Happiness	30	2.63 (0.67)	3.20 (0.55)	0.57 (0.31, 0.82)***	0.84

^*^p < 0.05, **p < 0.01, ***p < 0.001

^tt^*p* < 0.10

Results also showed large increases in happiness *(p* < 0.001, *d* = 0.84*)* and moderate increases in the importance of religion in participants’ lives (*p* = 0.02, *d* = 0.41) over the study period. The frequency of prayer in the last seven days increased a small amount (*p* = 0.053, *d* = 0.30), with 10 students reporting more frequent prayer or meditation at follow-up but four students reporting less.

Statistically significant improvements in mental health also were observed from baseline to follow-up and are shown in Table 4. Participants reported moderate improvements in reduced feelings of stress (*p* = 0.015, *d* = 0.41) and reduced anxiety and depression (*p* = 0.006, *d* = 0.49) in the last week. Problems sleeping were observed to decrease a moderate amount from baseline to follow-up (*p* = 0.02, *d* = 0.40).

**Table 4:** Changes in stress, anxiety, and sleep problems from baseline to follow-up

	*N*	Baseline Mean (*SD*)	Follow-up Mean (*SD*)	Mean change (95% CI)	*d*
Feeling stressed in last 7 days	31	2.39 (0.72)	2.06 (0.77)	0.32 (0.03, 0.61)*	0.41
Feeling anxious or depressed in last 7 days (PHQ4)	31	2.29 (0.66)	2.02 (0.68)	0.27 (0.07, 0.47)**	0.49
Problems sleeping in last 7 days	30	3.27 (0.98)	2.90 (1.01)	0.37 (0.02, 0.71)*	0.40

^*^p < 0.05, **p < 0.01, ***p < 0.001

### Qualitative Thematic Analyses

Qualitative data analysis revealed four main themes demonstrating how attention to spiritual health, especially in the digital messages, supported positive health and well-being for participants. Attention to spiritual health promoted: (1) Holistic health and self-care, (2) Positivity and mindfulness throughout the day, (3) Sharing the program with others, and (4) Cultural tailoring and engagement of Latina students.

The first theme highlighted participants’ appreciation of the intervention’s spiritual component and the focus on holistic and integrated aspects of health. The intervention messages reminded students about the importance of spiritual health: one student said that she was *“busy with college and would forget about my spirituality, so it’s nice to refocus back.”* (#12) Self-care behaviors were prompted by the holistic health messages and focus on wellness.

I like focusing on being fit like being healthy, like eating healthy, but I know it’s much more than that. It’s your mental health and also your spiritual health so it definitely changed my mindset of how I would better take care of myself that way. (#8)

I think [this program is] super important especially right now, you know, with things being really crazy. It is important to take care of yourself physically and spiritually, emotionally, all these different things. And health and wellness is really important and I feel like that is not talked a lot about in my culture. So I thought this [program] was such a great idea to get more Latina women involved in taking care of themselves. (#22)

The second theme revealed that the intervention and especially the digital messages encouraged positive feelings and mindfulness throughout the day. Several participants described how the messages helped them to begin the day feeling centered, motivated, and happy.

My favorite [text messages] were reflective ones that asked me to reflect on the people in my life or to meditate. The interpersonal ones really worked for me as I saw them when I first woke up and I carried them throughout the day. (#10)

A third theme related to how participants shared their participation in the program, and this encouraged reflective discussions with others.

I talked [to my sister] about how I want to work on health and wellness, and it opened a conversation about how she is going to go to therapy, and it opened up an honest conversation between us. … One message was about mindfulness that said to share with loved ones, so I got my family in on [being] mindful and being with them and in a community. (#29)

[Favorite messages were:] Take a moment one and the ones about exercising. I shared that you should always keep fun and joy in your life, walk with a friend, and sing loudly…. I thought my mom would stress so I would share and say maybe we should try them out. We did a lot of walking and going out and reflecting on ourselves. (#5)

The final theme revealed how the culturally tailored and holistic health program engaged Latina students. Participants identified with the Spanish terms and culturally sensitive images used in the digital messages and felt they were empowering and inspirational. One participant noted that the program was *“able to really intertwine cultural identity with wellness and health.”* (#31).

Some of [my favorite messages] were the ones that were in Spanglish, like small things bring you *felicidades* (happiness), and the messages that said to laugh, dance, sing. Little reminders that a healthy life is beyond working out and meal prep but also your happiness. Those inspirational quotes were really helpful. (#16)

## Discussion

This pilot study is the first to our knowledge that integrates spiritual messaging into a holistic digital health and wellness intervention for young adult Latinas. The combination of remote peer coaching and daily digital messages is a novel approach to health promotion, especially when used for primary prevention among a non-clinical but higher-risk population such as younger Latinas in the USA (Gershkowitz et al., 2021; Gonzalez et al., 2021). Given the limited number of faith- or technology-based interventions for Latino communities (Derose & Rodriguez, 2020; Gonzalez et al., 2021; Lancaster et al., 2014; Rew & Wong, 2006), *Examen Tu Salud* provides a practical example of how spiritual messaging content can be incorporated into digital health and wellness programs to engage, empower, and support participants’ health and well-being. Ignatian pedagogy informed intervention design in three main ways, including cultural tailoring to respond to the context and lived experience of participants, action and goal setting for personal growth and health and well-being, and reflection and evaluation of progress (Chubbuck, 2007; Silf, 2007; Traub, 2008). Participants in the 4-week program reported improvements in spiritual health, religiosity, and mental health outcomes; the largest changes were observed in spiritual well-being, connections to a higher power, and happiness. Notably, these pilot study results compare favorably to other faith-based interventions by demonstrating positive and medium effects on improvements in spiritual well-being and mental health (Goncalves et al., 2015; Marques 2022). Although these results must be viewed within limitations of the single group intervention study design and small sample, the intervention design and study results offer a novel, promising, and important example of health promotion for Latinas.

The *Examen Tu Salud* technology-based intervention was based on well-established theories of health behavior change and informed by Ignatian values, yielding a holistic health program and applying universal health promotion strategies that are appropriate for all persons. The intervention messages conceived of spiritual health practices through references to prayer along with meditation, mindfulness, yoga, and nature walks. Spirituality does not require connection to a specific religious belief (Campesino & Schwartz, 2006), and God or a higher power was not directly mentioned in intervention messaging to ensure program relevance for all participants. Instead prayer, gratitude, and nourishing one’s spirit were featured in messages. Most participants (58%) rarely attended religious services, but several studies with college students in the USA suggest that Latino students are highly likely to embrace spiritual perspectives and practices regardless of their attendance at organized religious services (Campesino et al., 2009; Morgan Consoli et al., 2018).

The qualitative data revealed that cultural tailoring and attention to spiritual and holistic health resonated with participants, making them feel calm, happy, empowered, and motivated for health improvement – findings that align with research showing that spiritual practices prompt positive emotions which help to alleviate stress (Hawks et al., 1995; Hunter-Hernandez et al., 2015; Koenig, 2012). Mental health improved among participants in the *Examen Tu Salud* study, who reported less stress, anxiety, and fewer problems sleeping after intervention participation. The combination of observed improvements in spiritual health, religiosity, happiness, and mental health suggests that the culturally tailored intervention was relevant, engaging, and potentially effective.

Although there are various ways to integrate spirituality into health curricula (Goncalves et al., 2017), overall, the field of health promotion has not paid sufficient attention to spiritual health and well-being. The World Health Organization (WHO) definition of health refers only to physical, mental, and social dimensions, despite multiple efforts to add spiritual health (Gerhardt-Strachan, 2022). Ignoring spiritual health and failing to recognize it as a collective asset may be particularly harmful to communities where it is central, such as among Latina women who shape the spiritual perspectives of their family (Campesino & Schwartz, 2009) or young adult Latinos who rely on spirituality for overcoming challenges and thriving in the university environment (Morgan Consoli et al., 2018). With more than 80% of the world’s population identifying as religious (Pew Research Center, 2012) and the robust evidence base linking spirituality to mental and physical health, it is important for health professionals to embrace spiritual health in health promotion programming.

## Limitations

Conclusions about program effectiveness and generalizability are limited due to the research design, brief intervention, and small sample size. The single group intervention study design weakens conclusions about program effectiveness, as there was not a control group to compare to changes observed in the intervention group. However, the novelty of a digital health intervention for this particular community using combined physical, mental, and spiritual health messaging warranted exploring the impact of such an intervention using a single group intervention study design. Further, although the program lasted only four weeks, this was viewed as acceptable and feasible for busy students. Participants completed the final survey one week after program completion and five weeks after completing the baseline survey, which provided a limited period for observation of any changes in outcomes. Longer-term follow-up data collection at 6 and 12 months would strengthen the findings and is planned for future program evaluation.

Lastly, only 34 participants were enrolled in the available time and with sufficient resources to provide weekly coaching to all participants. Although coaching was brief—averaging 30 min per week per participant—additional time was required for scheduling and note taking. Because coaches were students attending a graduate program, there were constraints on the number of study participants that could feasibly be enrolled and managed. Participants who were more engaged by the digital messages or completed the intervention may have been more likely to respond to the follow-up survey than withdrawn participants or those who were less engaged throughout the intervention. However, given the positive pilot study results and high follow-up survey response rate, a larger trial with a comparison group and extended follow-up period at 6 and 12 months is warranted.

## Conclusion

The *Examen Tu Salud* program demonstrates a novel digital approach for incorporating spiritual components into a health promotion program that is relevant for all people regardless of the type or presence of religious affiliation. Even with broad views of spirituality incorporated into messages, this pilot study observed significant improvements in measures of spiritual health, religiosity, and mental health outcomes. Young adult Latina participants valued the integrated and comprehensive program approach that encouraged physical, mental, and spiritual self-care in alignment with Ignatian values and pedagogies and prompted with evidence-based behavior change strategies. Caution is warranted in generalizing these results to other settings due to the limited study single group intervention study design, brief follow-up, and small sample size. However, the program model illustrates an intervention approach that institutes of higher education, faith-based organizations, and community settings should consider for holistic health promotion interventions.

## Appendix 1

### Spiritual Health Questions

#### Connection to the Divine or Transcendent (8 Items)

How much do you agree or disagree with the following statements?

(1 = strongly disagree to 5 = strongly agree).

1. I feel close to God or a Higher Power
2. My spirituality helps me get through bad times
3. Talking every day with God or a Higher Power is important
4. I feel grateful for the gifts in life
5. God/Higher Power is loving and kind
6. My religion or spirituality guides me to do what is right
7. I depend on God or a Higher Power to help me with my problems
8. Helping my family is an important part of my spirituality or religion

#### Spiritual Well-Being (3 Items)

How much do you agree or disagree with the following statements about your life?

(1 = strongly disagree to 5 = strongly agree).

1. I believe there is some real purpose for my life
2. I feel good about my future
3. I feel very fulfilled and satisfied with my life

#### Connections to Others (3 Items)

How much do you agree or disagree with the following statements about your relationships and the environment?

(1 = strongly disagree to 5 = strongly agree).

1. I maintain meaningful and fulfilling relationships with others
2. I have a good support network of caring people
3. I praise other people for their achievements

#### Connections to the Natural Environment (3 Items)

How much do you agree or disagree with the following statements about your relationships and the environment?

(1 = strongly disagree to 5 = strongly agree).

1. I engage in environmentally friendly behaviors, like conserving water, recycling, composting, using reusable water bottles, and taking public transportation when feasible
2. I take long walks or hikes to explore and appreciate the environment
3. I am aware of the earth’s natural resources and their respective limits, and that we need to conserve these resources

#### Religiosity

Over the past 7 days, how often did you pray or meditate alone?

(1 = nearly every day to 4 = not at all).

How important is religion in your life?

(1 = very important to 4 = not at all important).
